# Maxillary Tuberosity Fractures: A Structured Narrative Review and Clinical Decision-Making Considerations

**DOI:** 10.3390/healthcare14131983

**Published:** 2026-07-03

**Authors:** Marko Matijević, Lota Matijević, Petra Mikulić, Valentina Matijević

**Affiliations:** 1 Faculty of Dental Medicine, University of Rijeka, 51000 Rijeka, Croatia; oralnakirurgija.osijek@gmail.com; 2 Faculty of Dental Medicine and Health Osijek, University of Osijek, 31000 Osijek, Croatia; lotamat@gmail.com; 3 Private Dental Practice, 10290 Zaprešić, Croatia; matijevic5ra@gmail.com; 4 School of Medicine, Catholic University of Croatia, 10000 Zagreb, Croatia

**Keywords:** complications, extraction, fracture, maxilla, oroantral, sinus, tuberosity

## Abstract

**Background:** Maxillary tuberosity fracture is an uncommon but clinically significant complication of maxillary molar extraction. It may compromise immediate surgical outcomes, reduce posterior maxillary bone volume, and complicate subsequent prosthetic or implant rehabilitation. Despite its clinical relevance, the available literature remains limited and heterogeneous, and no universally accepted recommendations for diagnosis or management have been established. **Methods:** This structured narrative review was undertaken to synthesize clinically relevant evidence on anatomical risk factors, biomechanical mechanisms, diagnostic considerations, and management strategies associated with maxillary tuberosity fractures, with particular emphasis on biomechanical interpretation and intraoperative surgical decision-making. A targeted search of PubMed/MEDLINE and Scopus identified English-language publications relevant to the topic. After assessment for clinical relevance, 37 publications covering the period from 1967 to March 2026 were included in the final narrative synthesis. **Results:** The reviewed literature indicates that maxillary tuberosity fractures result from the interaction of anatomical and biomechanical factors, including maxillary sinus pneumatization, reduced posterior maxillary bone support, complex root morphology, and excessive or inappropriate force application during extraction. Early recognition of increased intraoperative resistance and timely modification of the surgical approach emerged as key factors in reducing structural complications. The available evidence also demonstrates substantial heterogeneity in reported management strategies and the absence of standardized clinical recommendations. Based on the narrative synthesis, a clinically oriented classification and decision-making framework are presented to support intraoperative assessment and surgical reasoning. **Conclusions:** Although the available evidence is derived predominantly from case reports, case series, retrospective studies, narrative reviews, anatomical studies, and selected surgical textbooks, the structured synthesis of this evidence provides a practical foundation for clinical decision-making. Improved recognition of biomechanical risk factors and structured intraoperative assessment may contribute to safer surgical management, preservation of posterior maxillary bone support, and maintenance of future prosthetic and implant rehabilitation options.

## 1. Introduction

Maxillary tuberosity fracture represents an uncommon but clinically significant complication of posterior maxillary tooth extraction, most frequently associated with maxillary molars. Despite its relatively low incidence, this complication remains clinically relevant because it may compromise not only the immediate surgical outcome but also the structural integrity of the posterior maxilla and the feasibility of subsequent prosthetic and implant rehabilitation [1,2].

The reported frequency ranges from approximately 0.15% to 5%, reflecting variability in study design, operator experience, and patient-specific anatomical conditions [1,3]. However, this complication should not be regarded as an incidental intraoperative event. Rather, it may be understood as a biomechanically influenced event occurring under anatomically and surgically unfavorable conditions. Advanced maxillary sinus pneumatization, reduced bone elasticity, and complex root morphology—including divergence, bulbosity, or hypercementosis—combined with increased surgical difficulty, as described in studies evaluating extraction-related complication risk and operative difficulty [3,4,5], may create a biomechanical environment in which force transmission during extraction exceeds the structural tolerance of the tuberosity.

From a surgical standpoint, maxillary tuberosity fracture represents a clinically important intraoperative event requiring prompt recognition and a shift from routine extraction to complication-oriented management. Clinical presentation may range from limited fractures involving small bone fragments to extensive disruptions affecting the posterior alveolar process and the maxillary sinus floor. Fragment mobility, fracture extent, and sinus involvement are among the principal factors influencing treatment selection. Inadequate management may result in oroantral communication, infection, delayed healing, neurosensory disturbances, and substantial loss of alveolar bone volume [6,7]. Such consequences may further complicate or even preclude future implant placement and prosthetic rehabilitation [8,9,10].

Despite its clinical importance, the currently available evidence remains limited and fragmented. Most published data are derived from case reports, retrospective analyses, and small case series [11,12,13,14], with considerable heterogeneity in reporting methods and treatment approaches. Consequently, no universally accepted classification system or standardized treatment protocol has been established.

Recent literature has also approached maxillary tuberosity fractures from educational and classification-oriented perspectives, reflecting increasing recognition of the need for more structured approaches to complication assessment and management [13,14].

In this context, the need is not for additional isolated reports, but for a practical synthesis that may assist intraoperative interpretation and surgical decision-making. However, despite recent conceptual and educational developments, clinically oriented interpretive approaches focused on biomechanical assessment and intraoperative surgical reasoning remain limited.

Unlike previous predominantly descriptive reports, this review integrates anatomical, biomechanical, and intraoperative considerations into a practical interpretive approach. This work was designed as a structured narrative review intended to support clinical reasoning rather than provide a formal systematic evaluation of evidence. The aim of this review is to provide a structured and clinically applicable analysis of maxillary tuberosity fractures, with particular emphasis on biomechanical interpretation and decision-making in surgical practice.

## 2. Materials and Methods

This study was conducted as a structured narrative review intended to synthesize clinically relevant evidence on maxillary tuberosity fractures, with particular emphasis on biomechanical interpretation and surgical decision-making.

Given the heterogeneity of the available literature and the predominance of case reports and small case series, this approach was considered more appropriate than a formal systematic review.

Relevant literature was identified through a targeted search of major biomedical databases, including PubMed/MEDLINE and Scopus. The literature search was conducted up to March 2026 and was limited to publications available in English. To improve transparency and reduce potential selection bias inherent to a narrative review design, the search and study selection process was performed in a structured manner. Particular emphasis was placed on methodological consistency and direct clinical relevance to maxillary tuberosity fractures.

The search process focused on publications related to maxillary tuberosity fractures, oroantral communication, posterior maxillary anatomy, surgical complications of tooth extraction, and clinical management strategies. Search terms included combinations of the following keywords: “maxillary tuberosity fracture”, “tooth extraction complications”, “oroantral communication”, “maxillary sinus”, “posterior maxilla”, “oral surgery complications”, and “surgical decision-making”. Relevant publications were screened, and studies considered most clinically relevant were included in the final narrative synthesis.

Study selection was guided primarily by clinical relevance and applicability to surgical decision-making. Case reports, case series, retrospective clinical studies, narrative reviews, anatomical studies, and selected surgical textbooks were considered eligible when they contributed directly to the clinical interpretation and management of maxillary tuberosity fractures.

Due to the limited and heterogeneous nature of the available evidence, formal systematic inclusion criteria and quantitative synthesis were not considered feasible. Accordingly, formal risk-of-bias assessment was not considered appropriate for the aims of this review.

Based on the narrative synthesis of the available literature, a clinically oriented classification and decision-making approach is presented to assist intraoperative interpretation and surgical reasoning. These concepts should be interpreted as pragmatic and interpretive clinical tools derived from the available literature and clinical experience rather than as validated systems or formal clinical guidelines.

## 3. Narrative Synthesis of Clinical Evidence

### 3.1. Anatomical and Surgical Considerations

The maxillary tuberosity represents a structurally complex anatomical region located posterior to the last maxillary molar, characterized by variable bone morphology, reduced cortical thickness, and close anatomical proximity to the maxillary sinus. Unlike other regions of the maxilla, the tuberosity often consists predominantly of low-density cancellous bone with limited cortical support, which directly influences its biomechanical behavior during tooth extraction and surgical manipulation [15,16].

A key anatomical determinant is the relationship between the tuberosity and the maxillary sinus. In many patients, sinus pneumatization extends posteriorly and inferiorly, resulting in thinning of the tuberosity and, in some cases, reduced separation between the alveolar process and the sinus floor, as previously described in studies evaluating post-extraction sinus expansion and posterior maxillary remodeling [17,18,19,20]. This configuration reduces structural stability and increases susceptibility to fracture under extraction forces [8,16,17,20].

Bone quality in the tuberosity region may be characterized by reduced mineral density and increased elasticity compared to other maxillary sites. While this may facilitate tooth extraction under controlled conditions, it also predisposes the region to uncontrolled fracture when excessive or improperly directed forces are applied. The tuberosity may therefore be regarded as anatomically susceptible to fracture, particularly during torsional or leverage-based extraction techniques [1,15].

Equally important is the relationship between root morphology and surrounding bone. Maxillary molars frequently present with divergent, bulbous, or hypercementosed roots, which may be intimately engaged within the trabecular bone of the tuberosity. This anatomical interlocking increases resistance to extraction and alters force distribution, resulting in transmission of extraction forces not only to the tooth but also to the surrounding bone structure [1,4,21].

In this context, tuberosity fracture can be interpreted as a biomechanical consequence of force exceeding the structural capacity of the bone–tooth complex [1,15].

Taken together, these anatomical characteristics make the maxillary tuberosity particularly susceptible to fracture, highlighting the importance of controlled surgical technique and careful force application during extraction procedures.

### 3.2. Incidence and Risk Factors

Maxillary tuberosity fracture should not be regarded as a random intraoperative complication, but rather as the consequence of interacting patient-related, tooth-related, and procedure-related factors that influence structural resistance during extraction.

Patient-related factors primarily reflect reduced structural resistance of the posterior maxilla. Anatomical conditions such as extensive maxillary sinus pneumatization and decreased bone density contribute to reduced structural resistance of the tuberosity during extraction [1,17]. Systemic conditions affecting bone quality may further increase susceptibility, although their role remains insufficiently defined [1].

Tooth-related factors are associated with root morphology and its mechanical interaction with surrounding bone. Divergent roots, bulbous apices, and hypercementosis increase retention within the alveolus and complicate controlled luxation. Ankylosis is particularly relevant because the absence of the periodontal ligament results in direct transmission of extraction forces from the tooth to the surrounding bone, thereby increasing the risk of uncontrolled fracture [1,11].

Procedure-related factors are likely the most important contributors to the development of tuberosity fractures. Recent complication-focused reviews have further emphasized the importance of surgical planning, force control, and intraoperative adaptability in minimizing extraction-related complications [22].

Excessive force application, inadequate surgical planning, and improper use of elevators or forceps may lead to uncontrolled stress distribution within a structurally compromised region. In particular, leverage-based techniques applied without prior root separation or adequate bone removal significantly increase the risk of fracture. Limited access, poor visualization, and underestimation of anatomical complexity further contribute to incorrect force direction and magnitude [1,2]. These observations are consistent with broader analyses of extraction-related complications emphasizing the role of operator judgment and technical adaptability in preventing adverse surgical outcomes [23].

In most cases, tuberosity fracture does not result from a single factor, but rather from the cumulative effect of multiple anatomical and surgical disadvantages.

In clinical practice, the decisive risk factor is not anatomy itself, but the misjudgment of anatomical resistance and inappropriate force application. Fractures most commonly occur when standard extraction techniques are applied to non-standard anatomical conditions without adequate modification of the surgical approach. Early recognition of resistance patterns and timely modification of technique remain key preventive strategies [1,2,11]. Recent predictive and complication-oriented studies have additionally highlighted the importance of individualized risk assessment and structured follow-up planning in reducing extraction-related complications [24].

Together, these factors form the basis for clinical classification and surgical decision-making.

### 3.3. Classification

Maxillary tuberosity fractures can be clinically classified based on fragment stability, size, and sinus involvement, as these factors strongly influence the appropriate management strategy [1,8,9]. Existing classification approaches, including recent educational and classification-oriented models, have primarily focused on descriptive categorization, whereas the present review focuses on clinical interpretation and biomechanical considerations relevant to surgical management [14].

The proposed classification is summarized in Table 1 and serves as a practical aid for the clinical considerations summarized in Table 2. It is derived from the synthesis of the available literature and should be interpreted as a pragmatic clinical approach rather than a formally validated classification system. Stable fractures (Type I–II) are generally managed conservatively, whereas unstable or displaced fractures (Type III) more often require surgical management.

### 3.4. Diagnosis

Diagnosis of maxillary tuberosity fracture is primarily based on intraoperative findings, as most cases are identified during tooth extraction rather than preoperatively. A sudden change in resistance, unexpected mobility of the alveolar segment, or displacement of the posterior maxillary bone during luxation should raise immediate suspicion of tuberosity involvement.

Clinically, the presence of excessive mobility of the maxillary tuberosity, often accompanied by attached soft tissue and, in some cases, the extracted tooth, represents a key diagnostic sign. Additional indicators may include bleeding from the maxillary sinus, communication with the sinus cavity, or visible displacement of the posterior alveolar segment [1,8,25].

Preoperative radiographic assessment may help identify potential risk factors, such as extensive sinus pneumatization, divergent roots, or reduced bone support; however, it cannot reliably predict the occurrence of fracture. Recent studies evaluating radiographic risk assessment and oroantral communication have further emphasized the importance of preoperative anatomical evaluation in posterior maxillary extractions [26,27]. When fracture is suspected or confirmed, further evaluation—particularly with CBCT—may be considered in selected cases to assess the extent of the fracture and the presence of oroantral communication, especially in complex cases [8,17].

Diagnosis should be regarded as a dynamic intraoperative process requiring continuous reassessment [1,2].

### 3.5. Clinical Decision-Making Algorithm

Management of maxillary tuberosity fractures should be based on careful assessment of key clinical variables, including fragment size, stability, displacement, and sinus involvement [1,8].

Two principal management approaches are most commonly described in the literature [1,8,28]:

(1) Preservation of the fragment when adequate stability is present;
(2) Removal of the fragment in cases of major displacement, instability, or associated complications.

Clinical decision-making should not rely solely on fragment size but should integrate multiple intraoperative findings, including fragment stability, displacement, and sinus involvement [1,2,29].

The clinical considerations summarized in Table 2 are intended to support intraoperative interpretation in conjunction with the proposed classification presented in Table 1. These concepts should be viewed as a clinically oriented interpretive approach rather than a prescriptive treatment algorithm or formal guideline.

**Figure 1:**
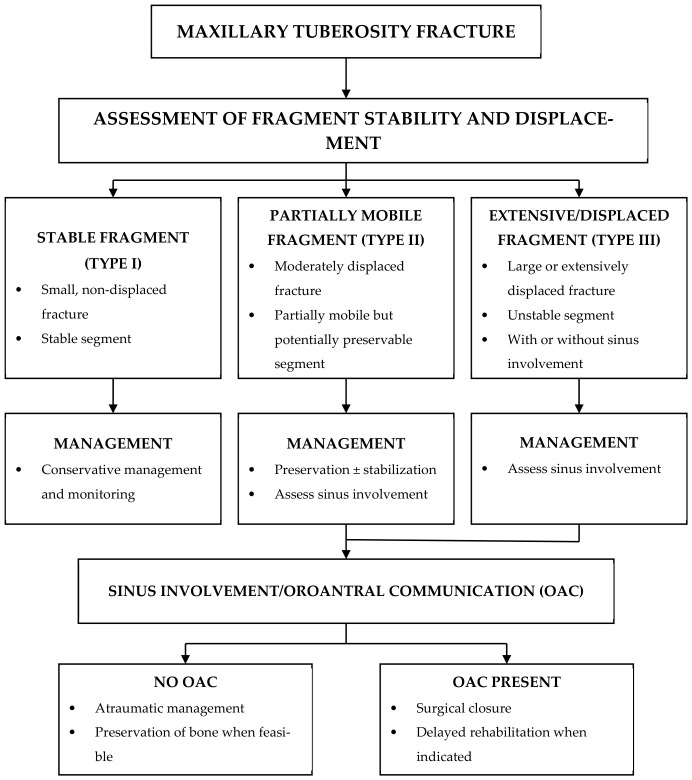
Clinical decision-making algorithm for maxillary tuberosity fractures based on fragment stability and sinus involvement.

The proposed classification (Table 1) and the clinical considerations summarized in Table 2 are complementary, with the classification providing structural categorization and the decision-making algorithm shown in Figure 1 guiding intraoperative management [1,9]. The decision-making framework is intended to support clinical reasoning and intraoperative judgment and should not be interpreted as a prescriptive or universally applicable guideline.

## 4. Discussion

### 4.1. Biomechanical Interpretation

Maxillary tuberosity fracture may be more appropriately understood as a clinically relevant complication reflecting the limitations of conventional extraction approaches under anatomically and biomechanically unfavorable conditions, as supported by existing clinical observations [1,8].

An important aspect of this review is the integration of currently available evidence into a practical framework for surgical decision-making. Although the reported incidence of maxillary tuberosity fracture remains relatively low, its consequences may be clinically significant, particularly in relation to postoperative rehabilitation and future prosthetic and implant rehabilitation. Despite this relevance, the available literature remains largely limited to case reports and small case series, which has contributed to the absence of standardized management principles and clinically oriented treatment frameworks [1,8].

Recent literature suggests that maxillary tuberosity fracture should not be regarded as an isolated technical complication, but rather as the consequence of interacting anatomical, biomechanical, and intraoperative factors. Classification-oriented and clinically focused reports have emphasized the continuing lack of standardized management principles and the importance of intraoperative interpretation during difficult posterior maxillary extractions [13,14]. In addition, clinical analyses have identified divergent root morphology, anatomical variability, and increased surgical difficulty as important contributors to fracture susceptibility, supporting the concept that fracture risk increases when extraction forces exceed the structural resistance of the posterior maxilla [1,11].

These observations further suggest that tuberosity fracture may reflect a biomechanical mismatch between applied extraction forces and the structural characteristics of the posterior maxilla, as described in implant and surgical biomechanics literature [15,17]. Clinical conditions such as ankylosis, hypercementosis, reduced periodontal ligament compliance, advanced sinus pneumatization, and diminished alveolar bone support may substantially alter the mechanical behavior of the tooth–bone complex. Under these circumstances, the tooth–bone complex loses part of its capacity to absorb extraction forces, which are then transmitted more directly to surrounding structures, predisposing the region to fracture, consistent with previously described anatomical and biomechanical considerations [1,15].

These findings are consistent with previously reported clinical observations, where anatomical constraints and sinus-related changes have been identified as key contributors to structural vulnerability in the posterior maxilla [17,18,19,20]. Furthermore, preservation of posterior maxillary bone volume has broader implications for future regenerative and implant therapy, including guided bone regeneration procedures and implant-supported rehabilitation in anatomically compromised posterior maxillary regions [30,31].

### 4.2. Intraoperative Resistance

Interpretation of intraoperative resistance represents one of the most important yet insufficiently discussed aspects of maxillary tuberosity fracture management. In routine clinical practice, resistance during luxation is often interpreted as incomplete mobilization, leading to escalation of force. However, this interpretation may be misleading in certain anatomical scenarios, as supported by studies addressing surgical difficulty and complication risk factors [2,11,32]. Resistance may instead represent a clinical warning sign indicating that further force application is not only ineffective but potentially harmful.

Recent analyses of surgical difficulty and complication prevention in oral surgery have emphasized the importance of intraoperative adaptability and controlled escalation of surgical intervention during difficult extractions [22]. Contemporary studies suggest that anatomical variability, operative complexity, and operator-related factors may substantially influence complication risk, particularly in demanding posterior maxillary procedures [2,11]. These observations further support the concept that resistance during extraction should not be interpreted solely as incomplete mobilization, but may instead represent a warning sign requiring modification of the surgical strategy.

The ability to recognize this transition point—where controlled extraction shifts toward structural failure—is a defining feature of surgical experience. Despite its clinical importance, this interpretative component is rarely formalized in the literature, which continues to focus predominantly on technical descriptions rather than decision-making processes. This gap between technical execution and intraoperative interpretation may represent an important limitation in current surgical practice and is indirectly reflected in complication analyses related to surgical technique and experience [2,23]. Preoperative imaging, including radiographic or CBCT evaluation, provides valuable anatomical information but remains inherently static and cannot reliably predict intraoperative mechanical behavior. Accordingly, surgical decision-making should remain dynamic and continuously adapt to tactile feedback, resistance patterns, and intraoperative findings.

### 4.3. Clinical Relevance

The absence of standardized clinical criteria is reflected in the marked heterogeneity of reported management strategies for maxillary tuberosity fractures. While most authors advocate preservation of the fractured segment whenever feasible and removal in cases of extensive displacement or oroantral communication [1,8,13], these recommendations are often descriptive rather than prescriptive. Clear thresholds defining when preservation is no longer viable remain insufficiently defined, leading to variability in clinical practice [13].

Recent systematic reviews and decision-oriented analyses focusing on oroantral communication management have further highlighted the considerable variability that still exists in the treatment of posterior maxillary complications. Contemporary literature emphasizes that management decisions are frequently influenced by defect size, sinus involvement, residual bone support, and long-term rehabilitation considerations rather than by universally accepted treatment thresholds [33,34]. Recent algorithm-based approaches have similarly underlined the importance of structured but flexible clinical reasoning in the management of oroantral defects and extraction-related posterior maxillary complications [35].

In this setting, the proposed classification and clinical considerations presented in this review may provide a useful interpretive aid in complex clinical situations [13,14]. Rather than imposing rigid rules, this approach organizes key variables—fragment stability, fracture extent, sinus involvement, and tooth condition—into a coherent clinical context that may assist surgical reasoning. Its value lies not in replacing surgical expertise, but in supporting more transparent and reproducible clinical interpretation.

An additional dimension that warrants greater emphasis is the long-term clinical significance of the maxillary tuberosity. Beyond its immediate role in extraction procedures, the tuberosity contributes substantially to posterior maxillary volume and serves as an important anatomical foundation for future implant placement. Studies evaluating post-extraction ridge alterations and sinus pneumatization have demonstrated that structural changes in the posterior maxilla may significantly influence long-term treatment outcomes, particularly in the context of implant planning [13,14,15]. Recent evidence has further suggested that complications involving oroantral communication and posterior maxillary bone loss may reduce the feasibility and predictability of subsequent implant rehabilitation, especially in patients with pre-existing bone deficiency [32]. This consideration extends the clinical relevance of tuberosity fractures beyond the immediate surgical event.

### 4.4. Prevention

Prevention of maxillary tuberosity fractures should be approached within a broader biomechanical and clinical framework rather than as a purely technical issue. Traditional recommendations emphasizing gentle force application are insufficient when considered in isolation. Prevention should instead be regarded as an anticipatory and adaptive process that begins with preoperative risk recognition and continues through intraoperative decision-making, as reflected in studies evaluating surgical difficulty and extraction-related complication risk factors [2,11,32].

This process includes early identification of unfavorable anatomical conditions, timely modification of surgical technique—such as tooth sectioning, controlled bone removal, or staged extraction—and a willingness to abandon conventional extraction approaches when intraoperative resistance indicates increased structural risk [2,11]. In this context, prevention is not a single action, but rather a continuous adjustment of surgical strategy based on anatomical conditions and intraoperative findings.

Recent literature addressing extraction-related complications and risk prediction has further emphasized the importance of individualized preoperative assessment and dynamic surgical planning in anatomically complex posterior maxillary procedures. Contemporary analyses have highlighted the relevance of sinus anatomy, root–sinus relationships, and radiographic risk evaluation in anticipating oroantral communication and structural complications during maxillary tooth extraction [26,27]. Additional complication-focused studies have also underlined the importance of patient-specific risk assessment, surgical adaptability, and structured follow-up strategies in minimizing adverse outcomes associated with difficult extractions [22,23,24,36].

Despite these insights, the current body of evidence remains inherently limited. The predominance of case reports and small case series restricts the ability to formulate standardized guidelines, while publication bias toward more severe or unusual cases may distort the perceived incidence and clinical significance of this complication. Furthermore, the relatively low frequency of tuberosity fractures presents a methodological challenge for large-scale prospective studies.

Future progress will likely depend on more systematic data collection, standardized reporting, and improved integration of clinical and radiographic findings, as emphasized in broader surgical and complication-related literature [11,12,23,24].

Given the limited availability of high-level evidence, this review should be interpreted as a structured synthesis of clinically relevant observations rather than a comprehensive systematic evaluation, with the primary aim of supporting practical clinical decision-making in oral surgical practice.

## 5. Clinical Implications

Maxillary tuberosity fracture should be regarded as a clinically important intraoperative complication requiring prompt reassessment of the extraction strategy rather than continuation of routine extraction techniques. In the presence of unexpected resistance, abnormal mobility of the posterior alveolar segment, or suspected structural instability, additional force application should generally be avoided and the surgical approach reconsidered to reduce the risk of further bone loss or extension of the fracture.

Preservation of the tuberosity fragment may be considered when adequate stability and soft-tissue attachment are maintained, whereas significant displacement, compromised structural integrity, or confirmed oroantral communication more often require surgical management, including fragment removal and appropriate closure procedures [1,8,13].

The proposed classification and clinical considerations may assist intraoperative judgment by integrating relevant clinical variables into a practical clinical framework. [13,14]. Rather than replacing surgical expertise, this approach is intended to support more consistent interpretation of fracture severity, structural stability, and sinus involvement during difficult posterior maxillary extractions.

From a broader clinical perspective, early recognition and appropriate management of maxillary tuberosity fractures remain important for preservation of posterior maxillary bone volume and maintenance of future rehabilitation options, particularly in implant and prosthetic treatment planning [17,37].

## 6. Limitations

Several limitations of this narrative review should be acknowledged. First, the currently available evidence on maxillary tuberosity fractures is derived predominantly from case reports, retrospective analyses, and small case series, which limits the strength of the conclusions and restricts the development of standardized evidence-based recommendations.

Second, considerable heterogeneity exists among published reports with regard to patient characteristics, anatomical conditions, fracture severity, and surgical management strategies. This variability limits direct comparability between studies and may influence interpretation of reported clinical outcomes. In addition, publication bias toward more complex or clinically significant cases may contribute to overrepresentation of severe presentations in the available literature.

Third, the proposed classification and decision-making framework are based on synthesis of the available literature and clinical reasoning rather than prospective clinical validation. Although intended to support intraoperative interpretation and clinical decision-making, the framework should be applied within the context of individual surgical judgment and specific clinical circumstances rather than as a universally applicable guideline.

Finally, the relatively low reported incidence of maxillary tuberosity fractures represents an important methodological challenge for future research, limiting the feasibility of large prospective studies and high-level evidence generation. Further progress will likely depend on more systematic data collection, standardized reporting, and improved integration of clinical and radiographic findings in future studies.

Accordingly, the present review should be interpreted as a structured clinical synthesis intended to support surgical decision-making rather than as a formal evidence-based guideline or comprehensive systematic evaluation.

In addition, some of the cited evidence originates from broader literature on extraction-related complications and posterior maxillary surgery rather than exclusively from studies specifically focused on maxillary tuberosity fractures.

## 7. Conclusions

Maxillary tuberosity fracture should not be regarded solely as a technical complication of tooth extraction, but rather as a clinically significant event influenced by anatomical conditions, biomechanical factors, and intraoperative decision-making. Its occurrence highlights the limitations of conventional extraction approaches in anatomically demanding posterior maxillary situations.

Accurate intraoperative interpretation of resistance represents an important element in reducing the risk of this complication, as resistance may indicate structural overload rather than incomplete tooth mobilization. Recognition of these biomechanical limitations may facilitate timely modification of the surgical approach and help prevent additional structural damage.

The proposed classification and decision-making considerations may support intraoperative assessment by integrating fragment stability, displacement, sinus involvement, and preservation potential into a practical clinical framework. However, these concepts are intended to support clinical reasoning and surgical judgment rather than function as definitive or universally applicable treatment guidelines.

Whenever feasible, preservation of the maxillary tuberosity should be considered because of its relevance for posterior maxillary bone preservation and future implant and prosthetic rehabilitation. Improved understanding of biomechanical risk factors and greater intraoperative awareness may contribute to safer surgical practice and more consistent treatment outcomes.

## Figures and Tables

**Table 1:** Proposed clinical classification of maxillary tuberosity fractures based on stability and displacement.

Type	Description	Clinical Characteristics	Management Implication
Type I	Small, non-displaced fracture	Stable segment without significant mobility	Conservative management and monitoring
Type II	Moderately displaced fracture	Partially mobile but viable fragment with preserved soft-tissue attachment and potential for stabilization	Conditional preservation and stabilization
Type III	Large or displaced fracture ± sinus involvement	Unstable segment with compromised structural integrity	Surgical management with fragment removal when indicated

**Table 2:** Key clinical parameters guiding decision-making in maxillary tuberosity fractures.

Clinical Parameter	Clinical Findings	Suggested Clinical Considerations	Potential Management Considerations
Fragment size and structural preservation potential	Small, non-displaced fragment	Structural preservation is generally feasible when adequate stability is present	Conservative management and monitoring
Fragment stability	Stable or partially mobile fragment with preserved soft-tissue attachment	Preservation may be considered if the fragment can be stabilized and sinus involvement is absent or limited	Preservation ± stabilization
Extent of displacement	Large or extensively displaced fragment	Structural integrity may be compromised, reducing the feasibility of preservation	Fragment removal with careful preservation of remaining bone support
Sinus involvement/oroantral communication	Presence of sinus communication or compromised sinus floor	Increased risk of postoperative complications and impaired healing	Surgical closure with consideration of delayed reconstruction when indicated
Combined intraoperative assessment including fragment–tooth relationship and feasibility of stabilization	Interaction of fragment mobility, displacement, sinus involvement, and remaining bone support	Clinical decision-making should be based on combined interpretation of intraoperative findings rather than isolated parameters	Individualized surgical management

## Data Availability

No new data were created in this study.
